# Gene discovery, gene expression and product manufacturing using an integrated fungal host system

**DOI:** 10.1155/S1463924603000117

**Published:** 2003

**Authors:** Richard P. Burlingame

**Affiliations:** Research & Development Dyadic International Inc. Jupiter FL USA


Journal of Automated Methods & Management in Chemistry
Vol. 25, No. 3 (May–June 2003) pp. 67–68

### Gene discovery, gene expression and product manufacturing using an integrated fungal host system†

**Richard P. Burlingame***
*Research & Development, Dyadic International, Inc., Jupiter, FL, USA*

Proteins are an increasingly important source of new products for numerous industries, including human and animal health, diagnostics, health and beauty aids, agricultural products, and chemicals. Because of the functional roles of proteins in living systems, proteins represent a large fraction of all biologically active molecules and the entire spectrum of biological activities. To exploit these properties to bring new products to these industries, there is an increasing demand for biological systems to discover, develop and manufacture proteins. Currently, there are a variety of systems available to perform discovery, development and manufacturing steps individually. However, some of these systems are limited with regards their abilities to discover and express genes from a large portion of the protein 'universe'. Others are impractical for commercial production of many proteins because of restrictive conditions necessary for growth and cultivation of the biological systems or the inability of those systems to produce proteins economically at large commercial scale.

Dyadic International is developing a system that allows gene discovery, gene expression, product development and manufacturing in a single biological host. This integrated system consists of gene discovery and gene expression components, both of which utilize the proprietary fungal host *Chrysosporium lucknowense*, or C1. C1 has differentiating advantages making it uniquely capable of functioning as an integrated discovery through manufacturing system. As a eukaryote, C1 is capable of faithfully expressing genes from virtually any source. Unlike bacteria, it is capable of splicing introns, interrupting sequences present in many eukaryotic genes. It is also capable of performing post-translational modifications characteristic of many eukaryotic proteins, for example glycosylation, with no evidence of the hyperglycosylation described for yeast. This allows access to portions of the biodiverse gene pool not accessible to other screening systems. Since greater than 90% of the Earth's species are eukaryotic by some estimates [1, 2], the C1 system will be able to discover genes and gene products that bacterial and yeast systems will not be able to.

C1 also has a culture morphology, unique among fungi, that confers the ability not only to grow in up to 384-well microtitre dishes, but also for those microvolume cultures to be robotically transferred from well to well for culture replication [3, 4]. Finally, C1 is capable of secreting and producing proteins under a variety of fermentation conditions at high yields, allowing cost-effective production of a large variety of proteins.

The gene-discovery engine utilizes robots for the preparation and assay of gene expression libraries in C1. The requisite genes can be from various sources—genomic or cDNA from individual organisms, DNA isolated from environmental samples, individual gene variant sets generated in the laboratory. These genes are cloned into expression vectors and introduced into C1. The unique morphology of C1, described above, leads to the formation of individual transferable elements, or propagules. The formation of these propagules results in non-viscous growth in culture, allowing adequate aeration in microtiter wells. In addition, formation of propagules results in lack of pelleting, surface matting and aerial sporulation. As shown in table 1, this eliminates canula tip clogging in robotic liquid-handling systems. The lack of aerial sporulation, in addition, prevents well-to-well cross-contamination.

Using a Zymark (Hopkinton, MA, USA) Allegro system, the propagules are transferred to the wells of microtitre dishes, each well containing on average one or a small number of cloned genes. After cultivation in the microtitre wells, the libraries of expressed genes are replicated, again using the Allegro robotic system. It is the lack of tip clogging and cross-contamination that makes the latter step possible.

The replicate, or daughter libraries, are then assayed for proteins or activities of interest. These can be enzymes, targets for fungicides, pharmaceutical targets or biotherapeutics. The assays are performed using a Zymark Staccato system, allowing both high throughput and flexibility in the assay protocol.

Once activities are found, clones expressing the activities are 'cherry-picked' from a second set of daughter plates and re-assayed to verify the activity. The genes of interest are isolated from individual clones expressing the activity. Since the method used for screening dictates that the gene is expressed at some level in C1, there is a high probability of success that the gene can be overexpressed to commercially viable levels. Numerous tools to optimize expression, secretion and host strain optimization have been developed [5] and continue to be developed. Using these tools, a number of native and heterologous genes have been overexpressed in C1 (table 2).

After a gene has been successfully overexpressed in the laboratory setting, versatile culture conditions

*e-mail: rburlingame@dyadic-group.com
†This paper was initially presented at the ISLAR 2002 Conference and is reproduced here by kind permission of Zymark Corporation.

*Table 1. Suitability of fungi for robotic screening.*

| Strain | Formation of propagules | Phenotype |
|---|---|---|
| *Chrysosporium lucknowense* C1 | +++++ | no tip clogging |
| Dyadic strain 2 | +++ | no tip clogging |
| Dyadic strain 3 | ++ | no tip clogging |
| One fungal species | ++ | no tip clogging, but some large pellets |
| Ten fungal species | – to ++ | tip clogging |

*Table 2. Examples of genes expressed in C1.*

| Gene product | Gene source | Level of expression |
|---|---|---|
| Endoglucanase 5 | C1 | 15 g/l |
| Glucoamylase | *Aspergillus* | 1 g/l |
| Interleukin 6 | human | 10 mg/l |
| TNF receptor analogue | human | 50 mg/l |

*Table 3. Fermentation parameters for C1 and other fungi.*

| | *Aspergillus* | *Trichoderma* | *C. lucknowense* C1 |
|---|---|---|---|
| pH | 3.0–6.0 | 3.0–6.0 | 4.5–9.0 |
| Temperature (°C) | 30–37 | 25–32 | 25–44 |
| Viscosity | 1500–2000 | 200–1000 | < 10 |
| Fermentation (days) | 8–9 | 6–7 | 5 |

(table 3) allow the products of genes expressed in C1 to be readily scaled up. The broad range of temperature and pH, the low culture viscosity, and short cycle time offer numerous options to develop processes to produce proteins economically that might otherwise be unstable or produced at lower yields in other production systems.

The C1 system is the first integrated system of its kind, allowing gene discovery, gene expression, process optimization and product manufacturing in a single host organism. That these processes can all take place in a single organism increases the likelihood of success and concurrently reduces the cycle time between conception and commercialization. Reduced probability of failure and shorter development timeline will facilitate development of numerous new industrial, pharmaceutical and consumer products in a timely and cost-effective manner.

### References

1. Bull, A. T., Goodfellow, M. and Slater, J. H., *Annual Review of Microbiology*, **46** (1992), 219.
2. Bank, D., *Wall Street Journal*, **22** (January 2002).
3. Emalfarb, M. A., Punt, P. J., van Zeijl, C. and van den Hondel, C., PCT Application WO 01/79558, 2001.
4. Emalfarb, M. A., PCT Application WO 01/25468, 2001.
5. Emalfarb, M. A., Burlingame, R. P., Olson, P. T., Sinitsyn, A. P., Parriche, M., Bousson, J. C., Pynnonen, C. M. and Punt, P.J., PCT Application WO 00/20555, 2000.

